# Transcriptome profiles of sturgeon lateral line electroreceptor and mechanoreceptor during regeneration

**DOI:** 10.1186/s12864-020-07293-4

**Published:** 2020-12-07

**Authors:** Jian Wang, Chengcheng Lu, Yifan Zhao, Zhijiao Tang, Jiakun Song, Chunxin Fan

**Affiliations:** 1 International Joint Center for Marine Biological Sciences Research, Ministry of Science and Technology, Shanghai Ocean University, Shanghai, China; 2 Key Laboratory of Exploration and Utilization of Aquatic Genetic Resources, Ministry of Education, Shanghai Ocean University, Shanghai, China; 3 Institute for Marine Biosystem and Neuroscience, International Center for Marine Studies, Shanghai Ocean University, Shanghai, China

**Keywords:** Mechanosensory, Electrosensory, Regeneration, Specification, Sturgeon

## Abstract

**Background:**

The electrosensory ampullary organs (AOs) and mechanosensory neuromasts (NMs) found in sturgeon and some other non-neopterygian fish or amphibians are both originated from lateral line placodes. However, these two sensory organs have characteristic morphological and physiological differences. The molecular mechanisms for the specification of AOs and NMs are not clearly understood.

**Results:**

We sequenced the transcriptome for neomycin treated sturgeon AOs and NMs in the early regeneration stages, and de novo assembled a sturgeon transcriptome. By comparing the gene expression differences among untreated AOs, NMs and general epithelia (EPs), we located some specific genes for these two sensory organs. In sturgeon lateral line, the voltage-gated calcium channels and voltage-gated potassium channels were predominant calcium and potassium channel subtypes, respectively. And by correlating gene expression with the regeneration process, we predicated several candidate key transcriptional regulation related genes might be involved in AOs and NMs regeneration.

**Conclusions:**

Genes with specific expression in the two lateral line sensory organs suggests their important roles in mechanoreceptor and electroreceptor formation. The candidate transcriptional regulation related genes may be important for mechano- and electro- receptor specification, in a “dosage-related” manner. These results suggested the molecular basis for specification of these two sensory organs in sturgeon.

## Background

Lateral line system is an ancient vertebrate sensory system in fishes and amphibians [1, 2]. Two different lateral line receptors, the electrosensory ampullary organs (AOs) and mechanosensory neuromasts (NMs), were found in non-neopterygian fish, including sturgeon, paddlefish and sharks, and some amphibians [3–8]. AOs enable fishes and amphibians to detect weak electric fields, including low-frequency membrane potentials and myogenic potentials that leak out of aquatic preys and predators [2, 3]. NMs respond to water displacement surrounding the body. Together, the electrosensory and mechanosensory divisions of lateral line system help these aquatic animals with detecting prey/predator, avoiding obstacle, intraspecific communication and other behaviors [2, 5].

A number of evidences support that both of AOs and NMs originate from lateral line placodes [5, 9–11]. NMs are formed by the central placodal zone, whereas AOs are formed by the lateral flanking zones [12]. The receptor cells of these two sensory organs possess distinct morphology. NM hair cells have a single kinocilium flanked by a stepped array of stereocilia called the ‘hair bundle’. Similar type of mechanosensitive hair cells also reside in the auditory and vestibular systems of the inner ear for all vertebrates including mammals [13, 14]. AOs electroreceptor cells of sturgeons have a single kinocilium, and are surrounded by the supporting cells with large numbers of long sterocilia. Similar structures are also found in other non-neopterygian [4, 11, 15]. NMs were lost in amniotes, however, similar type of mechanosensitive inner ear hair cells were kept for all vertebrates. AOs were also lost in some teleosts and amphibians and no analogous organs kept in most of higher vertebrates. For some teleosts, different types of electroreceptors evolved independently [2, 11]. The investigation about specification of AOs and NMs would help us understanding the origins and evolution of animal sensory system.

Although the AOs and NMs are both derived from lateral line placode, they show obvious morphological and physiological distinctions. Molecular mechanisms for these differences are not clearly understood. Several studies, including analyses of the sensory epithelium transcriptome of paddlefish, have identified some genes commonly expressed in both AOs and NMs, including *notch1, atoh1, eya1, eya4, parvalbumin-3, pou4f3* and so on [16–18]. However, the systemic transcriptome comparison between AOs and NMs was seldom reported.

In previous study, we found sensory receptor cells in AOs and NMs of Siberian sturgeon could be damaged by neomycin and regenerated in 7 days, and the cell proliferation were up-regulated at 12 h-post treatment (hpt) [15]. Investigations on gene expression during AOs and NMs regeneration could reveal molecular mechanisms for the formation of these two sensory organs. In this study, we sequenced the transcriptomes for neomycin treated sturgeon AOs and NMs in the early regeneration stages. By de novo assembling a sturgeon transcriptome and quantifying gene expression levels, we compared the gene expression between these two sensory organs. And by correlating gene expression with the regeneration process, we located several candidate key transcriptional regulation related genes in AOs and NMs regeneration.

## Results

### Sturgeon transcriptome de novo assembly and annotation

**Fig. 1:**
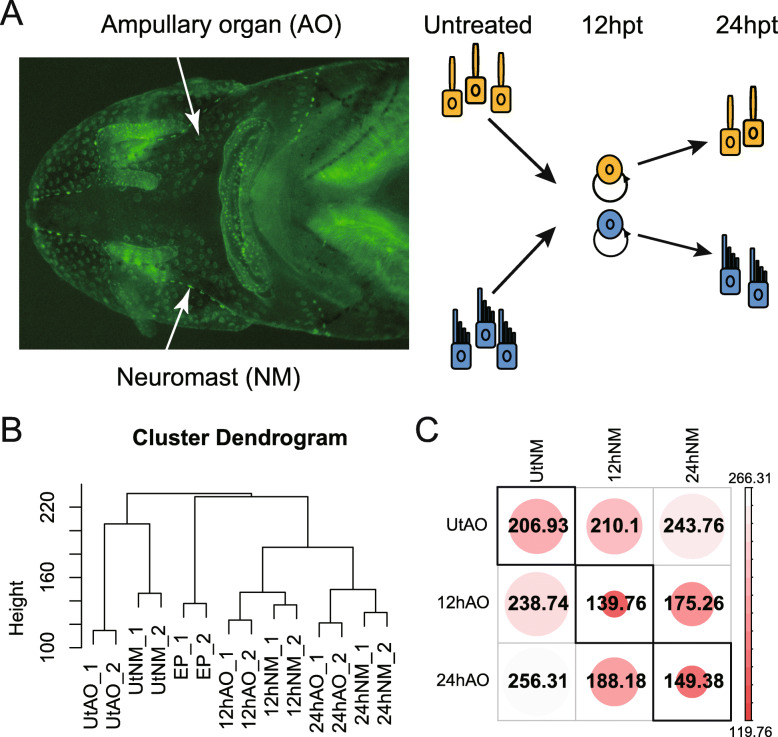
General gene expression profiles among transcriptome samples. **a** Illustration of two different sturgeon sensory organs under fluorescent stereomicroscope. Ampullary organs (AO) and neuromasts (NM) were dissected from neomycin treated sturgeon after 12 h and 24 h, as well as normal fish (Untreated). **b** Hierarchical clustering of all sequenced RNA samples. Numbers at the end of sample ID indicate two experimental repeats. Ut is short for sensory organs from untreated fish. EP is short for general epithelia dissected from ventral side of trunk. **c** Euclidean distances matrix of different groups. The smaller number indicates a more similar expression profiles between two groups

High quality RNAs were extracted (RINs > 8.0) from neomycin treated AOs and NMs in 12 hpt and 24 hpt, as well as untreated control AOs, NMs and general epithelia (EPs) (Fig. 1a) of Siberian sturgeon (*Acipenser baerii*), with each tissue has two replicated RNAs. Total 14 mRNA-Seq libraries were constructed for Illumina sequencing. Sequencing results were used to generate a de novo sturgeon transcriptome using procedures shown by supplementary figure 1. Total ~ 67 Gbp raw reads were obtained for 14 mRNA-Seq libraries. After quality control, total ~ 45 Gbp cleaned paired reads were used for assembling. Total 725,228 contigs were returned by Trinity (Table 1). Of these, 162,788 contigs had at least one ORF longer than 300 bps, and corresponding peptides were used for coding gene annotation.

**Table 1:** Summary of de nove transcriptome assembly and annotation

Items	Counts
De novo assembled contigs	725,228
Long-ORF (> 300 bp)contigs	162,788
Annotated transcripts	83,500
Unigenes	22,647

Predicated peptides were compared against protein sequences from Swiss-Prot and a close *Acipenser* relative, *Acipenser ruthenus* (sterlet), to identify orthologous genes. After combining orthologs to Swiss-Prot and sterlet proteins, we presented a sturgeon reference transcriptome including 83,500 transcripts belonging to 22,647 unigenes (Table 1). Nucleotide sequences and annotations have been uploaded to NCBI database (GEO accession: GSE151096). The average length of annotated contigs is around 1780 bp (Supplementary figure 2).

### Gene expression profiles of two types of sensory organs were most similar at 12 hpt during regeneration

Expressions of annotated genes were quantified by aligning cleaned reads to annotated transcripts and normalized by edgeR [19]. By clustering all sequenced samples based on Euclidean distance, we found high expression similarity between each experimental repeats (Fig. 1b). In general, all samples at 12 hpt and 24 hpt were relatively similar on expression. The EPs and untreated sensory organs were more different from others.

We also calculated the Euclidean distance between AOs and NMs sample groups particularly, based on gene expression change folds to EPs (Fig. 1c). The expression profiles of AOs and NMs were most similar at 12 hpt (distance = 139.76), and were most divergent for untreated samples (distance = 206.93).

In our previous study, we found that after damaged by neomycin, cell proliferation reached highest level at 12 hpt for both AOs and NMs. Both AOs and NMs sensory cell increased obviously at 24 hpt. And these damaged sensory cells recovery completely in 7 days [15]. Here, the overall sample expression profiles in this study were also consistent with phenotype features in the proliferation and differentiation process of two sensory organ types (Fig. 1a).

### Specifically expressed genes in two types of lateral line sensory organs

**Fig. 2:**
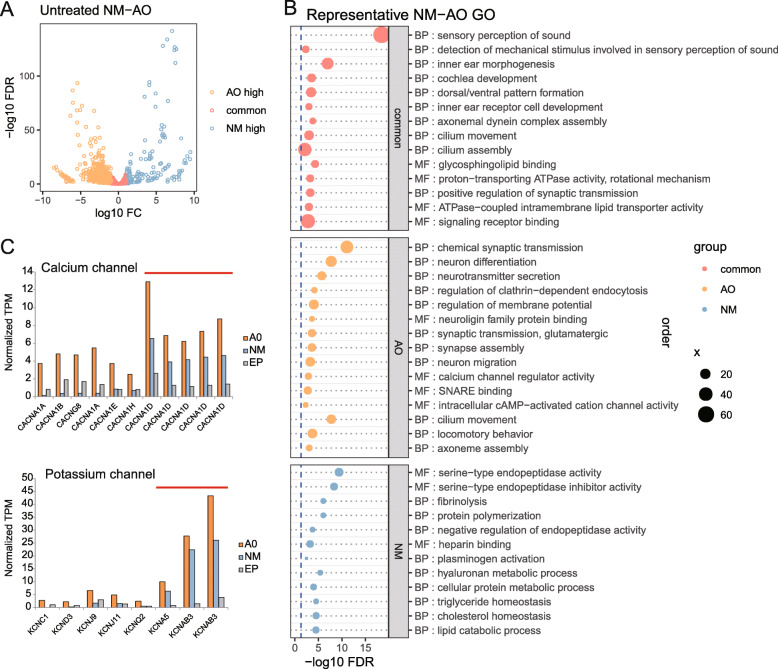
Specifically expressed genes in two types of untreated sensory organs compared to EPs and ion channel genes expression. **a** Volcano plot displays differentially and commonly expressed genes between AO and NM. **b** Representative enriched GOs in biological process (BP) and molecular function (MF) for three gene groups. **c** Expression of calcium channels (top) and potassium channels (bottom) encoding genes. Y axes are average TPM (transcripts per million) which has been TMM (trimmed mean of M-values) normalized among samples. Red lines indicate Ca_v_1.3, K_v_1.5 and K_v_β3 type channels

The NMs and AOs at stage 45 contain a number of mature receptor cells [4, 15]. To explore the lateral line sensory organs specifically expressed genes, differentially expressed genes among untreated tissue samples were investigated by edgeR. We found 2074 genes were highly expressed in lateral line sensory organs compared to EPs. Most of these (1418 genes) showed no significant expression difference between two sensory organs. More interestingly, 539 genes were significantly highly expressed in AOs, and 117 genes were significantly highly expressed in NM (Fig. 2a, Supplementary Table 1). We found some previously reported hair cells marker genes were detected both in AOs and NMs, including *cpv3* (parvalbumin-3, TRINITY_DN97159_c2_g2), *atoh1* (TRINITY_DN99639_c0_g1), *pou4f3* (TRINITY_DN100538_c0_g1), *six1* (TRINITY_DN104693_c4_g1, TRINITY_DN108998_c3_g1), *eya1* (TRINITY_DN116229_c3_g3, TRINITY_DN116229_c3_g4) and so on. In addition, some marker genes of presynaptic ribbon synapses, a special structure for sensory cells, including *ctbp2, rims2, otof* and *slc17a8* were also enriched in both AOs and NMs (Supplementary Table 1), most of which have more than one copies. These results also confirmed the reliability of our transcriptome assembly and quantification analysis.

The gene ontology (GO) enrichment analysis indicated that common genes most participated in acoustico-lateralis system related functions, including “sensory perception of sound”, “inner ear receptor cell development”, “cilium movement” and so on. AO specific genes were participating in functions related to morphogenesis and physiology of neural system and cilium (Fig. 2b), such as “neuron differentiation”, “chemical synaptic transmission”, “synapse assembly”, “regulation of membrane potential”, “axoneme assembly”, “cilium movement” and “locomotory behavior” and others. Whereas, the NMs specific genes were enriched mainly in protein lysis and polymerization, as well as specific lipid homeostasis, such as “serine endopeptidase activity”, “fibrinolysis” and “protein polymerization” (Fig. 2b).

### Predominant calcium and potassium channel encoding genes for sturgeon lateral line

Several calcium channels and potassium channels coding genes were found in our transcriptome assembly. All these ion channels coding genes were expressed higher in AOs than in NMs at different degrees. More than one copy of *cacna1d*, which encode the voltage-gated calcium channel subunit alpha (Ca_v_1.3) were abundantly expressed in sturgeon AOs. Some other voltage-gated calcium channel genes were also detected in AOs with obviously lower levels (Fig. 2c). The relatively predominate potassium channel genes were *kcnab3* and *kcna5,* which produce the voltage-gated potassium channel subunit beta-3 (K_v_-beta-3) and potassium voltage-gated channel subfamily A member 5 (K_v_1.5), respectively (Fig. 2c, Supplementary Table 1). They were also expressed abundantly in NMs, but with lower levels compared to AOs.

### Canonical Wnt signaling pathway was up-regulated in regeneration process

**Fig. 3:**
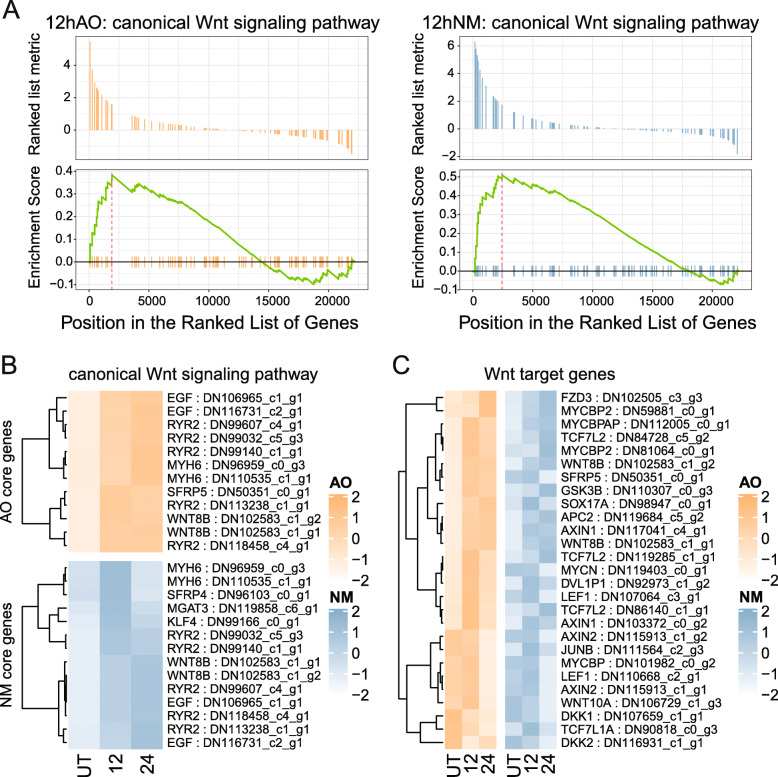
Expression profiles of genes in canonical Wnt signaling pathway during regeneration. **a** GSEA results indicate genes in canonical Wnt signaling pathway up-regulated at 12 hpt relative to untreated, for both AO (yellow, left) and NM (blue, right). **b** Heatmaps depict expression of core canonical Wnt signaling pathway genes from GSEA analyses during regeneration of AO (yellow, top) and NM (blue, bottom). Darker color represents higher relative expression level. **c** Expression of representative Wnt signaling target genes during regeneration of AO (yellow, left) and NM (blue, right). Darker color represents higher relative expression level

We have found cell proliferation of AOs was up-regulated at 12 hpt for neomycin treated sturgeon [15]. In this study, we further investigated the expression of genes which were involved in canonical Wnt signaling pathway, during regeneration process. According to Gene Set Enrichment Analysis (GSEA) analyses, genes in canonical Wnt signaling pathway was generally up-regulated at 12 hpt both for AOs (enrichment score: 0.383) and NMs (enrichment score: 0.510), compared to untreated samples (Fig. 3a). The up-regulated core genes for both AOs and NMs were also basically from same families including *wnt8*, *egf* (epidermal growth factor), *ryr* (Ryanodine receptor) (Fig. 3b). These genes were also up-regulated at 24 hpt compared to untreated samples for both two sensory organs in different degrees.

As shown in the heatmap, most of the Wnt target genes were expressed with relatively low levels in the untreated mature AOs and NMs. Most genes were up-regulated at 12 hpt when cell proliferation of the sensory organs were reaching to the peak, and decreased at 24 hpt when the sensory receptor cells started to differentiate (Fig. 3c).

### Candidate key transcriptional regulation related genes in AO and NM regeneration

**Fig. 4:**
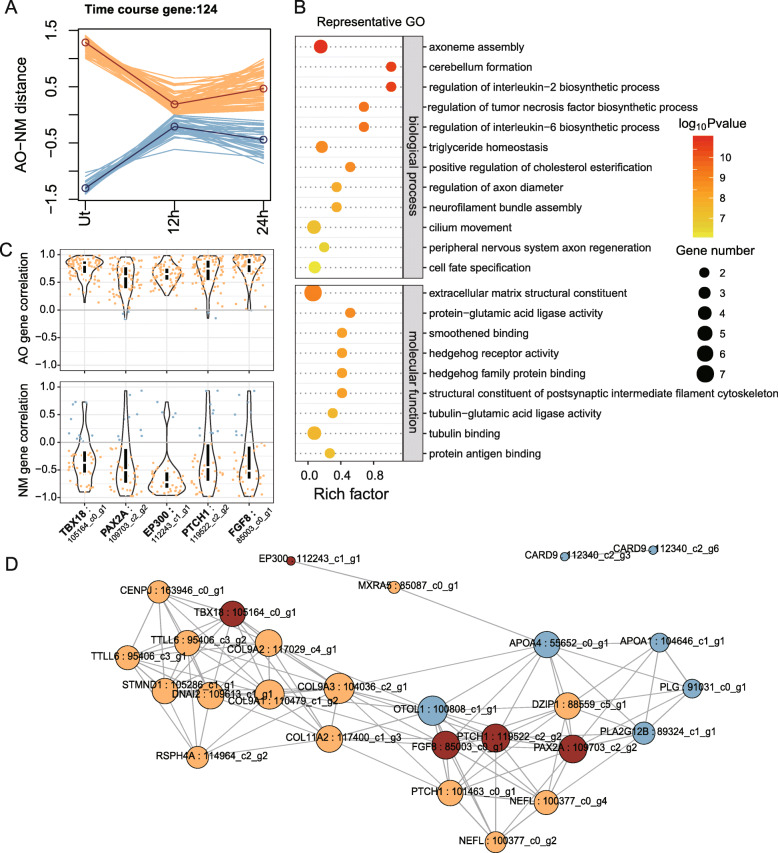
Genes with increasing expression differences between two sensory organs during regeneration time course. **a** X axis is regeneration stages. Y axis is scaled gene expression change fold of AO relative to NM. Genes of higher expression in untreated AO are colored in yellow, and higher expression genes in untreated NM are blue. Dark colored lines represent average of each gene sets. **b** Representative enriched GOs for the candidate gene set in plot A. **c** Violin plots of expression correlation to key genes involved in transcription regulation. Y axes are Pearson’s correlation coefficient to AO highly expressed genes (top) and NM highly expressed genes (bottom). Positive correlated AO high genes or negative correlated NM high genes are colored in yellow, and negative AO or positive NM genes are blue. The top and bottom sides of the black rectangles are the 3rd quartile and 1st quartile, and white lines are medians of all gene dots. **d** Co-expression network of closely correlated genes (*r*^2^ > 0.7) in representative GOs. Yellow and blue nodes represent genes highly expressed in AO or NM, respectively. Brown nodes are genes involved in transcription regulation and highly expressed in AO. The node diameter is proportional to sum of absolute value of correlation coefficients

During the regeneration process after neomycin treatment, the phenotypic differences of the two sensory organs also increased, until the formation of fully differentiated organs which could be partial reflected by the untreated samples in our study. We hypothesize that genes whose expression differences were increased along regeneration time course may play important roles in the fate determination of these two sensory organs, regardless of whether they were specifically enriched in the organs. Based on this, 124 candidate genes were identified whose expressions along regeneration had both of the two following features: 1) no significant differences between AOs and NMs at 12 hpt; 2) highest divergence between untreated AOs and NMs. Of these 124 genes, relative mRNA levels of 85 genes were gradually increased in AOs and 39 genes were increased in NMs (Fig. 4a). Representative enriched GOs of these 124 genes for “biological process” and “molecular function” were shown in Fig. 4b. Specific genes involved in these processes or enabling these activities were listed in supplementary Table 2. We found some GO terms related to nervous system regeneration were enriched mostly due to AOs high expression genes, such as “axoneme assembly”, “cerebellum formation”, “neurofilament bundle assembly”, “cilium movement”, “peripheral nervous system axon regeneration”, “hedgehog receptor activity” and “structural constituent of postsynaptic intermediate filament cytoskeleton”. Whereas, GO terms related to inflammatory reaction such as “regulation of interleukin-2 biosynthetic process”, “regulation of tumor necrosis factor biosynthetic process” and “triglyceride homeostasis” were enriched mainly based on NMs genes.

Of all these representative genes, we noticed five transcriptional regulation related genes. Their corresponding protein products are DNA binding transcription factors Pax2a, Tbx18, transcription cofactor Ep300, as well as Fgf8 and Ptch1 which are involved in signaling transduction activated by morphogens. A violin plot illustrated the Pearson’s correlation coefficients (r) of these five genes with the other 123 genes on expression (Fig. 4c). Expressions of these genes were highly correlated with most of others. Generally, they were positively correlated with most AO highly expressed genes (*r* > 0.610 for half AO genes), and negatively correlated with NM genes (*r* < − 0.339 for half NM genes). The *tbx18* and *fgf8* displayed strong correlation with half of AO high genes (*r* > 0.816 and *r* > 0.827, respectively), whereas half of NM genes have close negative correlation with EP300 (*r* < − 0.717). We also investigated pairwise gene expression correlation for representative GOs above. All gene pairs with determination coefficient (r^2^) above 0.7 were linked in the network diagram (Fig. 4d). Three genes, *pax2a*, *ptch1* and *fgf8*, were more closely associated. But *tbx18* and *ep300* were relatively independent with other two genes. Besides, *ep300* also has a moderate positive correlation with *card9* (TRINITY_DN112340_c2_g6, *r* = − 0.797. Not shown on Fig. 4d). We suspect these five candidates might be key transcriptional regulation related genes in AO and NM specification, either by influencing extracellular signal transduction or by affecting transcription efficiency directly.

### The expression and phylogenetic analyses of the key transcriptional regulation related genes

**Fig. 5:**
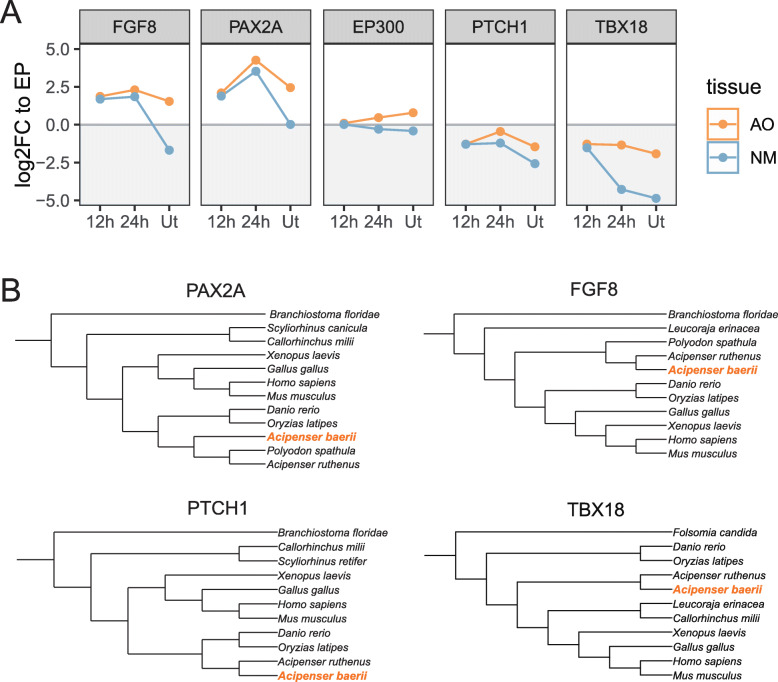
The expression and phylogenetic trees of candidate key genes. **a** Relative expression for five candidate genes during regeneration. X axis is regeneration stages. Y axis is expression change fold to EPs in log2 scale. **b** Phylogenetic trees were constructed using the ML method. Trees with the highest log likelihood were shown

We analyzed the relative expression for five transcriptional regulation related genes during regeneration, which were normalized to EPs. The expression levels of the five genes in AOs are significantly higher than NMs at untreated group, which suggests they could be used to distinguish mature AO and NM (Fig. 5a). Expressions of these genes relative to EPs were various. The *fgf8* and *pax2a* were highly expressed in AOs than EPs at all three time points. These two genes were also up-regulated in 12 hpt and 24 hpt in NMs, although they were not enriched in untreated NMs. The expression of *ptch1* and *tbx18* are lower in AOs and NMs than that in EPs. The *ep300* was moderately up-regulated in 24 hpt and untreated AOs, and down-regulated in NMs.

More remarkably, the expression differences between AO and NM for these five genes were dynamic during regeneration. There is no expression difference at 12 hpt between two sensory organs. However, the expression divergences were exhibited at 24 hpt. And even larger expression divergences were displayed between mature AOs and NMs.

Through transcriptome assembly, the complete CDSs of Siberian sturgeon (*Acipenser baerii*) *fgf8*, *tbx18* and *ptch1*, partial CDSs of *pax2a* and *ep300* were obtained. Based on amino acid sequences predicted (Supplementary Table 3), these Siberian sturgeon proteins/partial-proteins shared higher than 92 and 67% identities with chondrostean (sterlet and paddlefish) and human orthologs, respectively (Table 2). Furthermore, sequence alignment with orthologs suggested that over 95% of *pax2a* CDS was obtained. Unfortunately, most sequence of *ep300* is still missing*.* Phylogenetic trees of Fgf8, Tbx18, Ptch1 and Pax2a proteins were constructed using the ML method, with amphioxus or Collembola orthologs as outgroups. The sturgeon Fgf8, Tbx18, Ptch1 and Pax2a formed a cluster with other chondrostean orthologs, and separated with chondrichthyans, teleosts and tetrapods (Fig. 5b, Supplementary Table 4). These results indicate that these sturgeon genes are conserved with chondrostean orthologs.

**Table 2:** Protein sequence information of sturgeon and sequence similarity with corresponding orthologs

Protein	TRINITY gene id	Protein length (aa)	CDS type	Sequence similarity with orthologs				
				Species	Identity	Query Coverage^a^	NCBI Accession	Length (aa)
Fgf8	DN85003_c0_g1	211	complete	Sterlet	98.44%	90%	XP_034775828.1	193
				Paddlefish	92.34%	99%	ARW70855.1	198
				Zebrafish	85.78%	100%	NP_571356.2	210
				Human	86.29%	82%	NP_149353.1	244
Pax2a	DN109703_c2_g2	446	5′ partial	Sterlet	96.86%	55%	XP_034774454.1	270 partial
				Paddlefish	98.28%	52%	ADZ48384.1	284 partial
				Zebrafish	72.26%	100%	NP_571259.1	391
				Human	67.02%	100%	NP_003978.3	417
Tbx18^b^	DN105164_c0_g1	571	complete	Sterlet	93.35%	99%	XP_033860043.1	571
				Zebrafish	72.37%	100%	NP_705951.1	554
				Human	69.59%	99%	NP_001073977.1	607
Ep300^b^	DN112243_c1_g1	112	3′ partial	Sterlet	96.52%	100%	XP_034774298.1	270 partial
				Zebrafish	75.00%	78%	XP_009297682.1	2646
				Human	67.77%	100%	NP_001420.2	2414
Ptch1	DN119522_c2_g2	1458	complete	Sterlet	98.35%	100%	XP_033875282.2	1457
				Paddlefish	94.50%	28%	ABP96781.1	418 partial
				Zebrafish	75.23%	99%	NP_001292471.1	1475
				Human	80.40%	96%	NP_001077072.1	1446

^a^The sequence coverage of alignment in corresponding sturgeon protein. ^b^Sequences of paddlefish orthologs are not available

## Discussion

Two different types of lateral line system receptors were found for sturgeon, the electrosensory ampullary organs (AOs) and mechanosensory neuromasts (NMs), both of which originate from lateral line placodes. To better understand the molecular basis of differentiation for these two sensory organs, we compared the differentially expressed gene among mature AOs, NMs and general epithelia (EPs), and found some sensory organ specific genes. We also damaged sensory cells in AOs and NMs with neomycin, and compared the transcriptome of AOs and NMs in the early regeneration stages at the prospect of finding key genes for differentiation of two sensory organs. Our findings suggested the molecular basis for specification of electro- and mechano- receptor in sturgeon.

No high quality annotated Siberian sturgeon genome is public available yet and only a few transcriptome studies were reported [20–22]. In consideration of transcripts divergences are very often among different tissues, we made a de novo assembly and annotation of Siberian sturgeon lateral-line system and epithelium transcriptome as reference with strict QC criteria. The reference transcriptome sequences and gene quantifications could be publicly accessed (GEO accession: GSE151096). We believe these data would be valuable references for more related studies.

Gene names used in this article were cited directly from Swiss-Prot database targets of various species. Since the Siberian sturgeon was reported to be octoploid with more than 200 chromosomes [23], although we already use a relatively strict criteria to define orthologs (long ORF, at least 70% query has 50% identity), it is still possible that annotated genes were classified to be correct gene family but not the exact family members. Therefore, further evidences from evolution, function and expression are needed to verify our findings.

Recent studies suggested the crucial role of calcium and potassium channels as molecular basis of electroreception for sharks and skates, and evolutionary changes in ion channel structure facilitate sensory adaptation [24, 25]. Sharks and skates use the voltage-gated calcium channel Ca_v_1.3 to initiate cellular activity, however, they utilize voltage-gated potassium channels and calcium-activated potassium channel to modulate their electrosensory cell activity, respectively. In our AOs and NMs transcriptomes for sturgeon, several different subtypes of calcium channels encoding genes were found (Fig. 2c). In Siberian sturgeon, among several calcium and potassium channel subtypes, the voltage-gated calcium channel subunit alpha Ca_v_1.3 encoding gene (*cacna1d*) and voltage-gated potassium channel encoding genes (*kcna5, kcnab3*) have the highest mRNA levels in AOs (Fig. 2c). Besides, in paddlefish, which also belongs to chondrostean, all the three orthologous genes were detected to be enriched in AOs [18]. These indicate that the calcium and potassium channels for electroreception in Chondrostei are similar to that in shark rather than skate. The electroreceptor of sturgeon and shark is mainly used for predation, whereas skates also use it for intraspecies communication. The voltage-gated potassium channels are used for rapid vesicular release from elaborate ribbon synapses, which is adaptive for predation [24, 25].

Recent years, Backer et al. reported several works on AOs and NMs gene expression for paddlefish [16–18, 26], which is relative close to sturgeon phylogenetically. We found many consistencies between our study and theirs. Besides the ion channel encoding genes mentioned above, we found expressions of *cpv3, atoh1, notch1, jag1, fgf3, rims2, ctbp2, slc17a8, otof, sox1, myt1, eya1, six1b* and some other genes were enriched in lateral line receptors of sturgeons compared to EPs (Supplementary Table 1). These genes were also highly expressed in paddlefish lateral line system, and most were reported essential for hair cell formation in various species. For example, protein product of *cpv3* (Parvalbumin-3) was also reported as the major Ca^2+^ buffer in mechanosensory hair cells of the inner ear of bullfrog and chicken [27], which is also a marker gene of skate electrosensory and mechanosensory hair cells [10], as well as a marker of zebrafish inner ear and lateral line hair cells [28, 29]. The *atoh1* is required for zebrafish hair cell formation in both the inner ear and lateral line [30]. The *atoh1*and *pou4f3* are known as transcriptional regulators for the proper differentiation and/or survival of vestibular and auditory hair cells for mouse [31, 32], which were also expressed in both developing NMs and AOs for paddlefish [26]. The expression of these genes in AOs and NMs suggests their common critical role in mechanoreceptor and electroreceptor formation. The *ctbp2, rims2, otof* and *slc17a8* are important markers of presynaptic ribbon synapse, which is a special structure in sensory cells like electroreceptor, mechanoreceptor and photoreceptor [24, 26, 33]. This suggests these sensory organs share common structure and molecular components, although they carry out distinct function.

Embryonic development and regeneration share a number of common regulation pathways. During embryonic stage, both of the AOs and NMs are located in lateral line placodes and closed to each other, so it is hard to separate them. Our previous study suggested that the gene involving AOs and NMs development is also up-regulated during AOs and NMs regeneration [15]. Wnt signaling is a critical pathway for NMs development and regeneration [34, 35]. In this study, we found not only Wnt signaling components, but Wnt target genes were up-regulated at 12 hpt (Fig. 3) when proliferation of supporting cell reached to highest level in AOs and NMs [15]. Therefore, investigating the regeneration related genes of AOs and NMs is helpful to decipher the mechanism of these two organs differentiation.

From our transcriptome analyses, we found five candidate genes might be key transcriptional regulation related genes during AO and NM regeneration, including *pax2a, tbx18, fgf8, ep300* and *ptch1.* These five genes were seldom reported in ampullary organs for other species. However, in zebrafish NMs, *pax2a* is expressed in hair cells, *fgf8a* is expressed in mantle cells [34, 36]. Besides, *pax2a* was also expressed in the otic region in zebrafish embryo [37]. In addition, as transcription factors/cofactor or components of signaling transduction, these five genes have ever been reported involving otic morphogenesis through patterning and segmentation [38–43]. Specially, zebrafish *pax2a* was essential for hair cell development [44, 45]. Ep300 functions as histone acetyltransferase and regulates transcription via chromatin remodeling [46], which was also reported to participate in regulation of neuronal differentiation [47, 48].

The essential effects of the above five genes in cell differentiation and organ development also suggest their potential roles to participate in regulations of AOs and NMs regeneration and differentiation. Furthermore, more and more evidences have suggested that not only expression, but also the dynamic “appropriate dosage” of regulatory genes are much more crucial for normal development and differentiation. For example, differential levels of zebrafish Pax2a modulate precursor cells towards the otic placode and epibranchial placodes, respectively [49]. Fgf8 protein distributes in an anterior to posterior gradient to regulate the neocortex patterning [50]. Sonic Hedgehog acts in a graded manner to pattern the ventral neural tube, and different concentrations of Shh induce the formation of distinct neuronal subtypes [51–53]. Expression levels of *tbx18* were found temporally changed in the developing of somites [54, 55]. Since NMs and AOs derived from the central and flanking field of lateral line placode, respectively [12, 16], it is possible that morphogens and transcription factors presented a gradient along the placode as the regulators for AO and NM specification. Thus, these five candidates may also be involved in mechano- and electro-receptor differentiation in a “dosage-related” manner.

Furthermore, *ptch1*coding protein is an inhibitory receptor of Shh signaling [39, 53]. Both of *ptch1* and *fgf8* expression are positive correlated with most AOs high genes and negative correlated with NMs high genes (Fig. 4c), it suggests Shh and Fgf signaling plays distinct role in AOs and NMs specification, respectively. The dual signaling system here in lateral line sensory receptor regeneration is reminiscent of neural tube pattern induced by Shh and Bmp signaling [56]. As reported, *Pax* could be regulated by Shh signaling, and *Tbx* are targets of Fgf signaling [57, 58]. So, the fate of AOs and NMs is probably determined by Fgf and Shh signaling, which functions through transcription factors Tbx18 and Pax2a. More evidences are needed to test this hypothesis.

## Conclusions

Our study provided a de novo assembled and annotated transcriptome of Siberian sturgeon (*Acipenser baerii*). We located some specific genes of ampullary organs (AOs) and neuromasts (NMs) and predominately expressed ion channel encoding genes for sturgeon, which may take critical role in mechanoreceptor and electroreceptor formation. We also predicted several candidate key transcriptional regulation related genes might be important for AOs and NMs differentiation in a “dosage-related” manner through Fgf and Shh signaling transduction.

## Methods

### Tissue collection and RNA extraction

One day-post fertilization Siberian sturgeon (*Acipenser baerii*) embryos were bought from Dalian Yongxin Sturgeon Development Company, and raised to stage 45 (10 day-post hatching) in artificial fresh water (63 mg CaSO_4_, 10 mg MgSO_4_, 4 mg KCl, 1.1 mg NaH_2_PO_4_ per liter of dH_2_O) at 18-20 °C. To obtain adequate RNAs for following sequencing library construction (at least 1 μg each library), corresponding tissues at each control and experimental condition were collected from 15 larvae and pooled together, respectively. Total 45 larvae were divided into 3 groups randomly (*n* = 15 each). For untreated control group, larvae were incubated with 0.02% 2-amino-benzoic acid ethyl ester (MS222; Sigma) for 10 min until immersion anaesthesia. Then, sensory epithelia of neuromasts (NMs) and ampullary organs (AOs) at the ventral side of head, and general epithelia (EPs) at the ventral side of trunk were separated under fluorescence stereomicroscope using dissecting knife after visualized with 0.005% DASPEI. For the other two groups, larvae were treated with 200 μM neomycin for 1 h to ablate the sensory cells in NMs and AOs as described previously [15], then NMs and AOs were separated at 12 h-post treatment (hpt) and 24 hpt respectively as above after larvae were anaesthetized. Dissected EPs, NMs and AOs at each control and experimental condition from multiple larvae were pooled together respectively, and each pooled sample was divided into two parts for RNA extraction. Total RNAs of each sample were extracted using TRIzol reagent (Invitrogen) according to manufacturer instructions.

### RNA-Seq library construction and sequencing

The quantity of total RNA was determined using a Qubit fluorometer (Life Technologies). The quality of RNA was assessed by measuring RINs using Bioanalyzer Chip RNA 7500 series II (Agilent). One microgram of total RNA from each sample was used to prepare an mRNA-Seq library with TruSeq™ RNA Sample Prep Kit (Illumina), following the manufacturer’s instructions. Library quality control was performed with a Bioanalyzer Chip DNA High Sensitive (Agilent). Each library had an insert size of 300–400 bps, and 2 X 109 bps paired-end sequences were generated using Hiseq 1500 (Illumina).

### De novo transcriptome assembly and annotation

As illustrated by the flowchart (Figure S1), RNA-Seq reads were cleaned by Trimmomatic [59] to remove Illumina adapters. The first 13 bases were cut off from the start of reads. Bases below Q3 were cut off if at the beginning or end of a read. Reads were scanned from 5′ end with a 40-bases wide sliding window, and rest bases on 3’end were discarded when the average base quality below Q30. Reads were discarded if below 70 bases in length. Only paired reads were used in the following assembling and mapping procedure. Cleaned reads from all tissues were pooled and assembled a de novo transcriptome by Trinity (v2.6.6) with default parameters.

Open reading frames (ORF) and corresponding peptides were predicted by TransDecoder for contigs. Only peptides longer than 100 aa were kept in the following annotation. Orthologs of long peptides to Swiss-Prot proteins (ftp://ftp.uniprot.org/pub/databases/uniprot/current_release/knowledgebase/complete/uniprot_sprot.fasta.gz) were obtained using NCBI blastp and high quality orthologs (query coverage above 70% and identity above 50%) were kept. The sterlet proteome was downloaded from NCBI (ftp://ftp.ncbi.nlm.nih.gov/genomes/all/GCA/004/119/895/GCA_004119895.1_ASM411989v1/). Sturgeon orthologs to sterlet were done by OrthoMCL [60] using default option (identity > 50% and E-value < 10^− 5^). Orthologs to Swiss-Prot and sterlet are merged together to get a reference Sturgeon transcriptome.

### Gene quantification, sample expression comparison and identification of differentially expressed genes

Cleaned reads of each sample were aligned to the annotated contigs following RSEM procedure in Trinity, to get gene counts and TMM (trimmed mean of M-values) normalized TPM (Transcripts per million). Pairwise Euclidean distances between samples were calculated based on TMM normalized gene expression, then sample hierarchical cluster was generated by R function hclust(). The gene expression change folds between tissue groups were detected by edgeR [19] using Fisher’s exact test model. The genes with more than 2-fold changes in expression (absolute value of log_2_FC > 1 and FDR < 0.01) between groups were considered as differentially expressed genes. Pairwise Euclidean distances between different tissue groups were calculated based on relative gene expression levels to EPs (log_2_FC to EPs) from edgeR results.

### Identification of genes with increased expression divergence between two organs during regeneration

Gene expression fold-change of AOs to NMs for every time point were calculated by edgeR. During regeneration course, genes with increased expression divergence between two organs were considered to be correlated with two organ differentiation. Genes were defined as having increased expression divergence when they meet the following requirements at the same time: 1) Genes had significant expression difference (absolute value of log_2_FC > 1 and FDR < 0.01) between untreated AOs and NMs; 2) genes had no difference at 12 hpt between two organs; 3) The fold-change between AOs and NMs at 24 hpt was smaller than that between untreated organs; 4) Gene expression levels in untreated AOs and 24 hpt AOs were either both higher than corresponding NMs at these two time points, or both lower than corresponding NMs.

Pairwise gene expression correlation coefficients (r) were calculated using R function cor(), based on relative gene expression levels to EPs (log_2_FC to EPs) from edgeR results. Gene pairs with *r*^2^ > 0.7 were considered as closely correlated genes. The R package igraph (https://igraph.org/r/) was used to generate co-expression network.

### Gene ontology (GO) enrichment and gene set enrichment analysis (GSEA)

GO annotation database of Swiss-Prot genes was downloaded from UniProt website. GOs of sturgeon transcripts were obtained according to annotation of Swiss-Prot targets. Enrichment analysis of target genesets were done by in-house R scripts using phyper() as the core function, built on that target gene number within the geneset is in hypergeometric distribution with all annotated genes as background. GSEA analysis was done by R package clusterProfiler [61]. Functions of genesets were classified by the annotation to GO biological process.

### Phylogenetic analysis

Protein sequences of Sturgeon gene were predicted by TransDecoder. Sequence identities of Sturgeon proteins and orthologs were obtained by blastp. Peptide sequences of orthologs were downloaded from NCBI (Supplementary Table 4). Gene trees were estimated by MEGA-X using Maximum likelihood (ML) method. After model test, the substitution model with the lowest BIC scores was selected to generate the ML phylogenetic tree for each gene. The trees were subsequently visualized and annotated by iTOL (https://itol.embl.de/).

## Supplementary Information

**Additional file 1.**

**Additional file 2.**

## Data Availability

The raw sequences and reference transcriptome as well as other processed data in this study could be accessed by GEO (GSE151096) on the National Center for Biotechnology Information (NCBI) website.

## Abbreviations

AO: Ampullary organ
NM: Neuromast
EPs: General epithelia
hpt: hour-post treatment
RIN: RNA Integrity Number
ORF: Open reading frame
NCBI: National Center for Biotechnology Information
GEO: Gene Expression Omnibus
GO: Gene Ontology
GSEA: Gene Set Enrichment Analysis
QC: Quality Control
RSEM: RNA-Seq by Expectation-Maximization
FC: Fold of change
FDR: False Discovery Rate

## References

[1] Schlosser G (2002). Development and evolution of lateral line placodes in amphibians. – II. Evolutionary diversification. Zoology..

[2] Crampton WGR (2019). Electroreception, electrogenesis and electric signal evolution. J Fish Biol.

[3] Teeter JH, Szamier RB, Bennett MVL (1980). Ampullary electroreceptors in the sturgeonScaphirhynchus platorynchus (rafinesque). J Comp Physiol.

[4] Gibbs MA, Northcutt RG (2004). Development of the lateral line system in the shovelnose sturgeon. Brain Behav Evol.

[5] Modrell MS, Bemis WE, Northcutt RG, Davis MC, Baker CVH (2011). Electrosensory ampullary organs are derived from lateral line placodes in bony fishes. Nat Commun.

[6] Freitas R, Zhang G, Albert JS, Evans DH, Cohn MJ (2006). Developmental origin of shark electrosensory organs. Evol Dev.

[7] Northcutt RG, Catania KC, Criley BB (1994). Development of lateral line organs in the axolotl. J Comp Neurol.

[8] Torroba M, Barrutia MG, Zapata AG (1991). Fine structure and histochemistry of the ampullary organ of the urodele amphibian Pleurodeles. Tissue Cell.

[9] Northcutt RG, Brändle K, Fritzsch B (1995). Electroreceptors and Mechanosensory lateral line organs Arise from single Placodes in axolotls. Dev Biol.

[10] Gillis JA, Modrell MS, Northcutt RG, Catania KC, Luer CA, Baker CVH (2012). Electrosensory ampullary organs are derived from lateral line placodes in cartilaginous fishes. Development..

[11] Baker CVH, Modrell MS, Gillis JA (2013). The evolution and development of vertebrate lateral line electroreceptors. J Exp Biol.

[12] Northcutt RG (1997). Evolution of Gnathostome lateral line ontogenies. Brain Behav Evol.

[13] McPherson DR (2018). Sensory hair cells: an introduction to structure and physiology. Integr Comp Biol.

[14] Gillespie PG, Müller U (2009). Mechanotransduction by hair cells: models, molecules, and mechanisms. Cell..

[15] Fan C, Zou S, Wang J, Zhang B, Song J (2016). Neomycin damage and regeneration of hair cells in both mechanoreceptor and electroreceptor lateral line organs of the larval Siberian sturgeon (Acipenser baerii). J Comp Neurol.

[16] Modrell MS, Baker CVH (2012). Evolution of electrosensory ampullary organs: conservation of Eya4 expression during lateral line development in jawed vertebrates. Evol Dev.

[17] Modrell MS, Tidswell ORA, Baker CVH (2017). Notch and Fgf signaling during electrosensory versus mechanosensory lateral line organ development in a non-teleost ray-finned fish. Dev Biol.

[18] Modrell MS, Lyne M, Carr AR, Zakon HH, Buckley D, Campbell AS, Davis MC, Micklem G, Baker CVH (2017). Insights into electrosensory organ development, physiology and evolution from a lateral line-enriched transcriptome. eLife..

[19] Robinson M, McCarthy D, Smyth G (2010). edgeR: a Bioconductor package for differential expression analysis of digital gene expression data. Bioinformatics (Oxford, England).

[20] Song W, Jiang K, Zhang F, Lin Y, Ma L (2015). Transcriptome sequencing, De novo assembly and differential gene expression analysis of the early development of Acipenser baeri. PLoS One.

[21] Vidotto M, Grapputo A, Boscari E, Barbisan F, Coppe A, Grandi G, Kumar A, Congiu L (2013). Transcriptome sequencing and de novo annotation of the critically endangered Adriatic sturgeon. BMC Genomics.

[22] Yue H, Li C, Du H, Zhang S, Wei Q (2015). Sequencing and De novo assembly of the gonadal Transcriptome of the endangered Chinese sturgeon (Acipenser sinensis). PLoS One.

[23] Birstein VJ, Poletaev AI, Goncharov BF (1993). DNA content in eurasian sturgeon species determined by flow cytometry. Cytometry..

[24] Bellono NW, Leitch DB, Julius D (2018). Molecular tuning of electroreception in sharks and skates. Nature..

[25] Bellono NW, Leitch DB, Julius D (2017). Molecular basis of ancestral vertebrate electroreception. Nature..

[26] Baker CVH, Modrell MS (2018). Insights into electroreceptor development and evolution from molecular comparisons with hair cells. Integr Comp Biol.

[27] Heller S, Bell AM, Denis CS, Choe Y, Hudspeth AJ (2002). Parvalbumin 3 is an abundant Ca2+ buffer in hair cells. J Assoc Res Otolaryngol.

[28] Hsiao C-D, Tsai W-Y, Tsai H-J (2002). Isolation and expression of two zebrafish homologues of parvalbumin genes related to chicken CPV3 and mammalian oncomodulin. Mech Dev.

[29] McDermott BM, Asai Y, Baucom JM, Jani SD, Castellanos Y, Gomez G, McClintock JM, Starr CJ, Hudspeth AJ (2010). Transgenic labeling of hair cells in the zebrafish acousticolateralis system. Gene Expr Patterns.

[30] Millimaki BB, Sweet EM, Dhason MS, Riley BB (2007). Zebrafish atoh1 genes: classic proneural activity in the inner ear and regulation by Fgf and notch. Development..

[31] Bermingham NA, Hassan BA, Price SD, Vollrath MA, Ben-Arie N, Eatock RA, Bellen HJ, Lysakowski A, Zoghbi HY (1999). Math1: an essential gene for the generation of inner ear hair cells. Science..

[32] Xiang M, Gao WQ, Hasson T, Shin JJ (1998). Requirement for Brn-3c in maturation and survival, but not in fate determination of inner ear hair cells. Development..

[33] Camacho S, Ostos MDV, Llorente JI, Sanz A, García M, Domezain A, Carmona R (2007). Structural characteristics and development of Ampullary organs in Acipenser naccarii. Anat Rec.

[34] Jiang L, Romero-Carvajal A, Haug JS, Seidel CW, Piotrowski T (2014). Gene-expression analysis of hair cell regeneration in the zebrafish lateral line. Proc Natl Acad Sci.

[35] Wada H, Ghysen A, Asakawa K, Abe G, Ishitani T, Kawakami K (2013). Wnt/Dkk negative feedback regulates sensory organ size in Zebrafish. Curr Biol.

[36] Lush ME, Diaz DC, Koenecke N, Baek S, Boldt H, St Peter MK, Gaitan-Escudero T, Romero-Carvajal A, Busch-Nentwich EM, Perera AG, Hall KE, Peak A, Haug JS, Piotrowski T (2019). scRNA-Seq reveals distinct stem cell populations that drive hair cell regeneration after loss of Fgf and Notch signaling. eLife.

[37] Pfeffer PL, Gerster T, Lun K, Brand M, Busslinger M (1998). Characterization of three novel members of the zebrafish Pax2/5/8 family: dependency of Pax5 and Pax8 expression on the Pax2.1 (noi) function. Development..

[38] Naiche LA, Harrelson Z, Kelly RG, Papaioannou VE (2005). T-Box Genes in Vertebrate Development. Annu Rev Genet.

[39] Chen Y, Struhl G (1996). Dual roles for patched in sequestering and transducing hedgehog. Cell..

[40] Torres M, Gomez-Pardo E, Gruss P (1996). Pax2 contributes to inner ear patterning and optic nerve trajectory. Development..

[41] Léger S, Brand M (2002). Fgf8 and Fgf3 are required for zebrafish ear placode induction, maintenance and inner ear patterning. Mech Dev.

[42] Trowe M-O, Maier H, Schweizer M, Kispert A (2008). Deafness in mice lacking the T-box transcription factor Tbx18 in otic fibrocytes. Development..

[43] Zarei S, Zarei K, Fritzsch B, Elliott KL (2017). Sonic hedgehog antagonists reduce size and alter patterning of the frog inner ear. Dev Neurobiol.

[44] Riley BB, Chiang M, Farmer L, Heck R (1999). The deltaA gene of zebrafish mediates lateral inhibition of hair cells in the inner ear and is regulated by pax2.1. Development..

[45] Whitfield TT, Riley BB, Chiang M-Y, Phillips B (2002). Development of the zebrafish inner ear. Dev Dyn.

[46] Tropberger P, Pott S, Keller C, Kamieniarz-Gdula K, Caron M, Richter F, Li G, Mittler G, Liu Edison T, Bühler M, Margueron R, Schneider R (2013). Regulation of transcription through acetylation of H3K122 on the lateral surface of the histone Octamer. Cell..

[47] Li X, Chen X, Zhou W, Ji S, Li X, Li G, Liu G, Wang F, Hao A (2017). Effect of melatonin on neuronal differentiation requires CBP/p300-mediated acetylation of histone H3 lysine 14. Neuroscience..

[48] Choi M, Ko SY, Lee IY, Wang SE, Lee SH, Oh DH, Kim Y-S, Son H (2014). Carbamylated erythropoietin promotes neurite outgrowth and neuronal spine formation in association with CBP/p300. Biochem Biophys Res Commun.

[49] McCarroll MN, Lewis ZR, Culbertson MD, Martin BL, Kimelman D, Nechiporuk AV (2012). Graded levels of Pax2a and Pax8 regulate cell differentiation during sensory placode formation. Development..

[50] Toyoda R, Assimacopoulos S, Wilcoxon J, Taylor A, Feldman P, Suzuki-Hirano A, Shimogori T, Grove EA (2010). FGF8 acts as a classic diffusible morphogen to pattern the neocortex. Development..

[51] Marti E, Bumcrot DA, Takada R, McMahon AP (1995). Requirement of 19K form of sonic hedgehog for induction of distinct ventral cell types in CNS explants. Nature..

[52] Briscoe J (2009). Making a grade: sonic hedgehog signalling and the control of neural cell fate. EMBO J.

[53] Dessaud E, Yang LL, Hill K, Cox B, Ulloa F, Ribeiro A, Mynett A, Novitch BG, Briscoe J (2007). Interpretation of the sonic hedgehog morphogen gradient by a temporal adaptation mechanism. Nature..

[54] Bussen M, Petry M, Schuster-Gossler K, Leitges M, Gossler A, Kispert A (2004). The T-box transcription factor Tbx18 maintains the separation of anterior and posterior somite compartments. Genes Dev.

[55] Kraus F, Haenig B, Kispert A (2001). Cloning and expression analysis of the mouse T-box gene Tbx18. Mech Dev.

[56] Tanabe Y, Jessell TM (1996). Diversity and pattern in the developing spinal cord. Science..

[57] Cairns DM, Sato ME, Lee PG, Lassar AB, Zeng L (2008). A gradient of Shh establishes mutually repressing somitic cell fates induced by Nkx3.2 and Pax3. Dev Biol.

[58] Isaac A, Cohn MJ, Ashby P, Ataliotis P, Spicer DB, Cooke J, Tickle C (2000). FGF and genes encoding transcription factors in early limb specification. Mech Dev.

[59] Bolger AM, Lohse M, Usadel B (2014). Trimmomatic: a flexible trimmer for Illumina sequence data. Bioinformatics (Oxford, England).

[60] Li L, Stoeckert CJ, Roos DS (2003). OrthoMCL: identification of Ortholog groups for eukaryotic genomes. Genome Res.

[61] Yu G, Wang LG, Han Y, He QY (2012). clusterProfiler: an R package for comparing biological themes among gene clusters. OMICS.

