# Supplementary figures and images for: Transcriptome profiles of sturgeon lateral line electroreceptor and mechanoreceptor during regeneration
Source: BMC Genomics. 2020 Dec 7;21:875. doi: 10.1186/s12864-020-07293-4 (PMC7720607; doi:10.1186/s12864-020-07293-4)

**Figure S1**

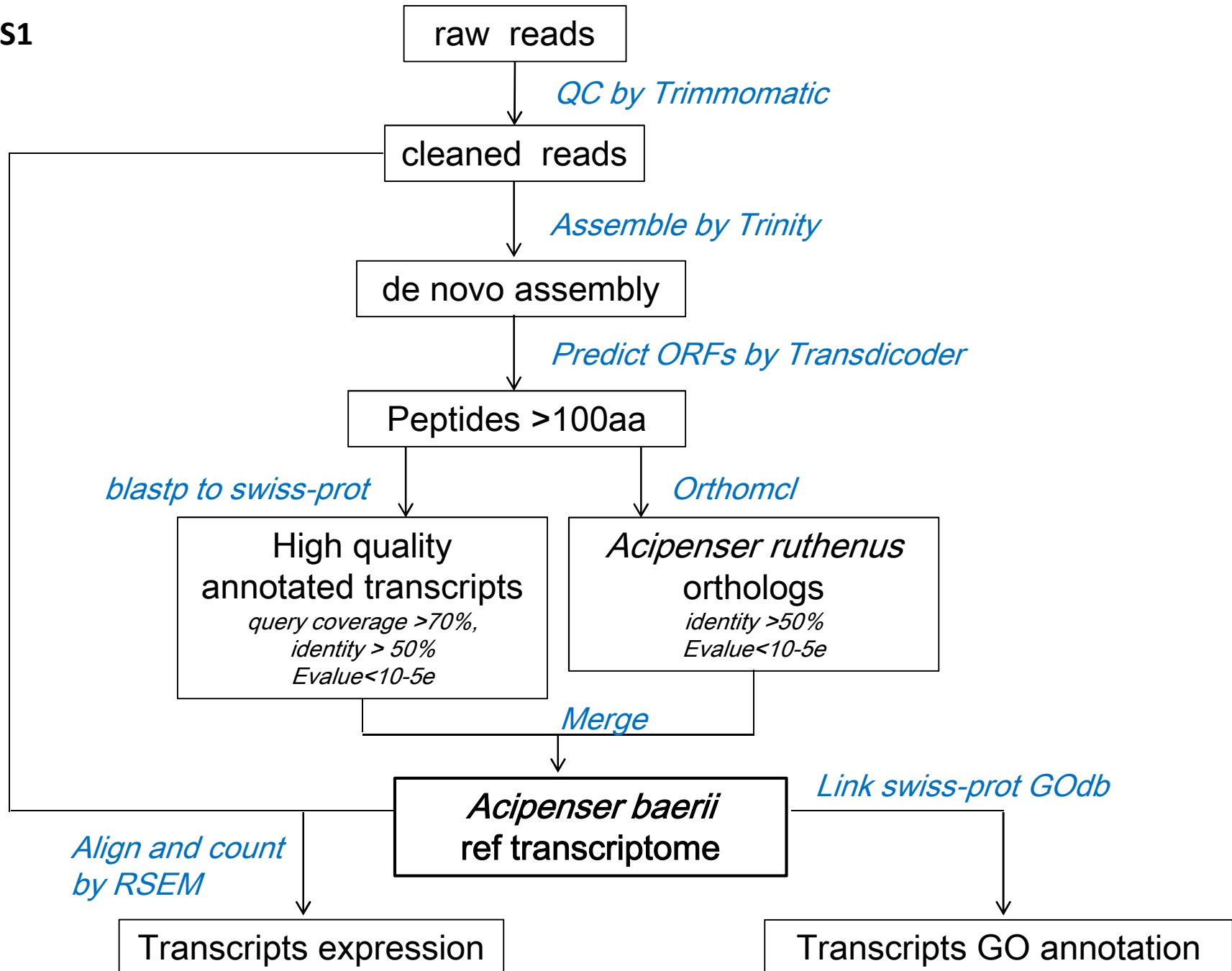

Figure S2

Statistics of annotated sturgeon transcriptome

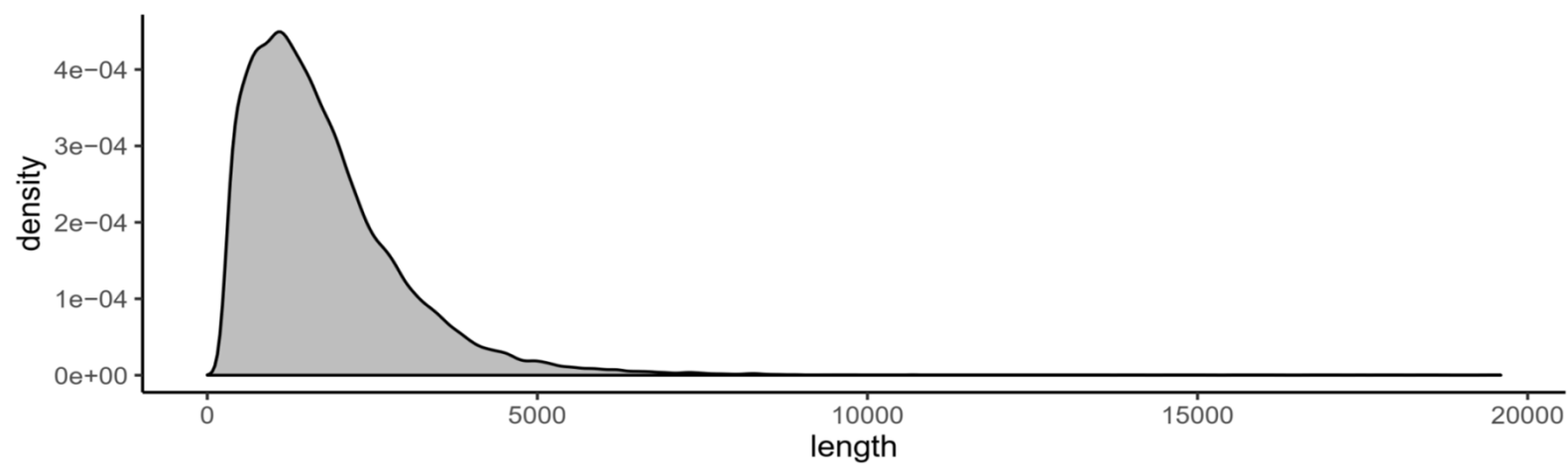

Supplement: Supplementary file 1 — Additional file 1. [file 12864_2020_7293_MOESM1_ESM.pdf]
